# Postoperative tibial plateau angle changes and their influence on ground reaction forces 6 months after TPLO: a prospective study

**DOI:** 10.3389/fvets.2024.1506848

**Published:** 2025-01-06

**Authors:** Frederik Volz, Daniela Eberle, Matthias Kornmayer, Julius Klever, Andrea Meyer-Lindenberg

**Affiliations:** ^1^LMU Small Animal Clinic, Centre for Clinical Veterinary Medicine, Ludwig-Maximilians-Universität München, Munich, Germany; ^2^Small Animal Clinic Stuttgart Plieningen, Stuttgart, Germany; ^3^Royal Veterinary College, University of London, London, United Kingdom

**Keywords:** cranial cruciate ligament disease, tibial plateau leveling osteotomy, tibial plateau angle, gait analysis, dog

## Abstract

**Objectives:**

The objectives of the study were to investigate the association between change in postoperative (post-op) tibial plateau angle (TPA) in dogs and cranial cruciate ligament disease (CCLD) after tibial plateau leveling osteotomy (TPLO) during 6 months on the post-op outcome.

**Methods:**

The inclusion criteria included 60 dogs with CCLD treated with TPLO fulfilled. TPA measurements were taken immediately post-op and 6 months post-op by three observers, and change in TPA was calculated. The outcome was evaluated using lameness score, owner questionnaire, and gait analysis performed preoperatively and 6 months post-op.

**Results:**

The mean change in TPA was 0.22 ± 0.75°. The interobserver reliability for TPA measurements was excellent. No differences in TPA measurements between observers were found (*p* = 0.07–0.105). No association between the change in TPA and outcome was found. The multivariate linear regression model for the symmetry index of peak vertical force (SIPVF) 6 months post-op was significant (*R*^2^ = 0.210; *p* = 0.031), and the TPA at 6 months post-op was the only significant factor (*ß* = 0.459; 95% CI: 0.41–1.44; *p* < 0.001), indicating that a lower TPA 6 months post-op results in lower SIPVF values.

**Conclusion:**

The study indicated that lower TPAs 6 months post-op lead to a more symmetrical gait in hindlimbs 6 months post-op. No other significant factor between post-op changes in TPA and outcome after TPLO was found. Our results showed little post-op TPA change up to 6 months. This indicates that change in TPA is not present as reported.

## 1 Introduction

**Figure 1:**
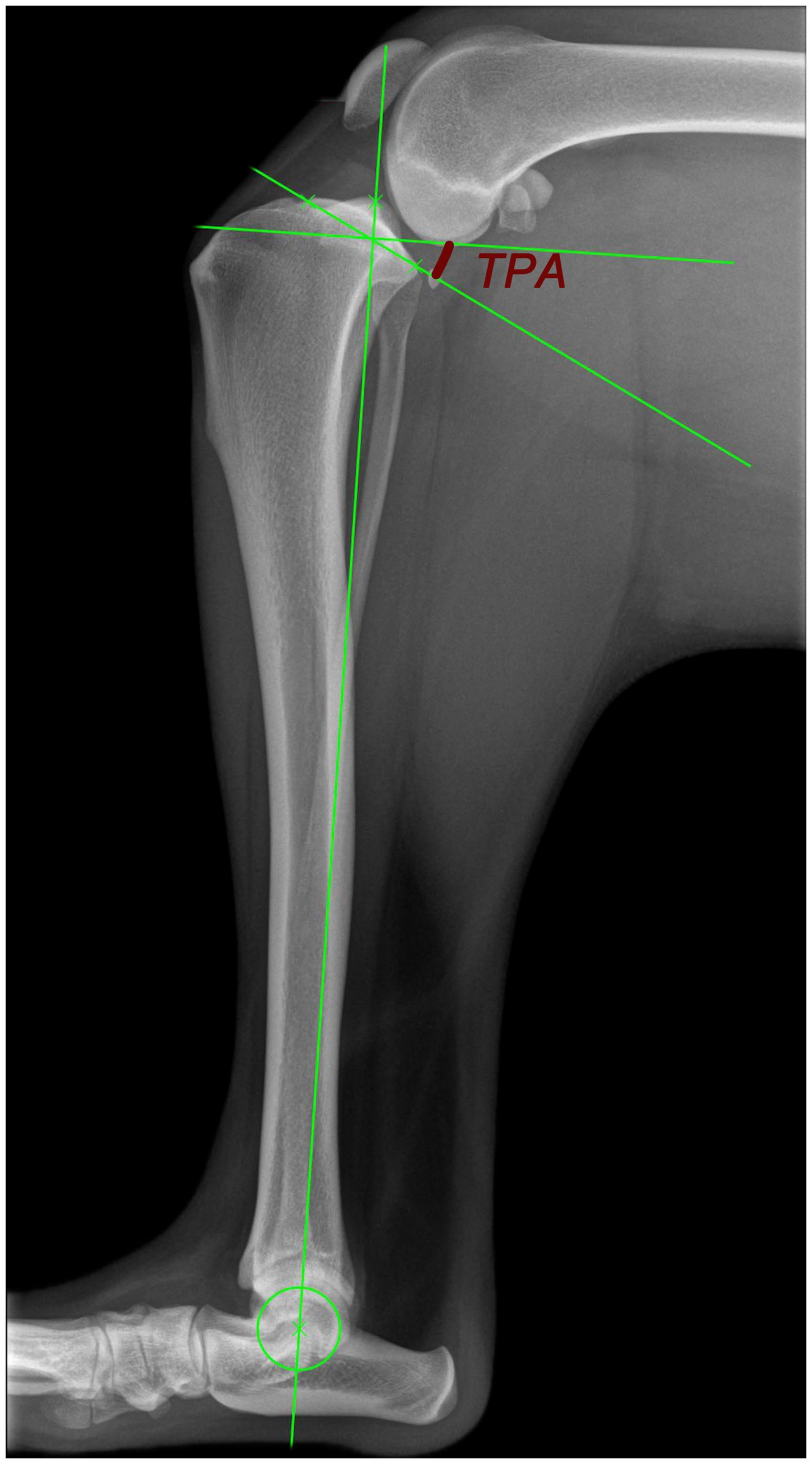
Radiograph for the measurement of the TPA according to Slocum and Devine (2). The mechanical tibial axis is a line from the center of the talocrural joint to the midpoint between the medial and lateral intercondylar tubercles. The TPA is the angle between the slope of the medial condyle perpendicular to the mechanical tibial axis (red angle line).

In 1993, Slocum and Slocum (1) described a surgical technique for the treatment of cranial cruciate ligament disease in dogs, the tibial plateau leveling osteotomy (TPLO). The goal of the TPLO was to eliminate cranial tibial thrust, the cranial displacement of the tibia that occurs with weight-bearing in the absence of an intact cranial cruciate ligament (1, 2). The rationale behind TPLO is that a post-operative tibial plateau angle (TPA) of 5–6 degrees minimizes or eliminates cranial tibial thrust, stabilizing the CCL-deficient stifle while weight-bearing (1). In veterinary medicine, the TPA is most often measured on two-dimensional radiographs (3). The tibial plateau angle (TPA) is defined as the angle between the slope of the medial tibial condyle and a line perpendicular to the mechanical axis of the tibia. The mechanical axis is established by a line extending from the center of the talocrural joint to the midpoint between the medial and lateral intercondylar tubercles (2) (Figure 1). Several studies demonstrated that the clinical outcome for dogs with cranial cruciate ligament disease (CCLD) treated with tibial plateau leveling osteotomy (TPLO) is superior to outcomes for those treated with tibial tuberosity advancement, the modified Maquet procedure, capsular-fascial imbrication, or lateral extracapsular suture (4–8).

In 2006, Moeller et al. (9) performed a retrospective study to evaluate the change in TPA during healing after a TPLO. They found a mean change in TPA of 1.5° by 65 days after TPLO and found no significant factor attributing this change in TPA (9). It was shown that the change in TPA after TPLO was significantly less and the osteotomy site healed faster when using locking screws compared to conventional screws (10). In contrast, another study found no significant difference between the change in TPAs using a non-locked plate and a locking-hybrid plate (11). Despite no significant difference in TPA change, they found that the tibial plateau rotation was higher and better maintained when using the locked-hybrid plate (11). In a series of Labrador Retrievers, it was shown that a postoperative TPA between 0° and 14° has no effect on the outcome measured between 4 and 17 months postoperatively with ground reaction forces (12). However, one retrospective study demonstrated that a lower TPA at reexamination between 42 and 84 days postoperatively was significantly correlated with improved weight-bearing, as measured using a stance analyzer (13). In addition, the aforementioned study also evaluated the effect of surgeon, experience, meniscal injury, initial TPA, direct postoperative TPA, and complications, which revealed that these variables had no impact on the outcome (13). Therefore, the clinical impact of the change in TPA on outcome remains unclear.

The purpose of this study was to (1) confirm the results by Wilson et al. (13) when using a treadmill gait analysis and (2) to evaluate the association between the change in TPA and clinical outcome 6 months postoperatively measured using subjective lameness, owner questionnaires, and objective treadmill-based force plate gait analysis during a walk. We hypothesized that (1) the change in TPA and the postoperative TPA is associated with outcome variables [lameness, ground reaction forces, Liverpool OsteoArthritis in Dogs (LOAD)] and (2) dogs with less change in TPA would have an association with improved ground reaction forces (GRF) up to 6 months postoperatively.

## 2 Materials and methods

Dogs were part of a prospective study at a veterinary teaching hospital between August 2019 and August 2022 (14). In the aforementioned study, some dogs received an intraarticular injection of hyaluronic acid (HA) (*n* = 21), platelet-rich plasma (PRP) (*n* = 21), or no intraarticular injection (*n* = 20), but all received a TPLO due to CCLD. The injection of HA (2 mL of Hyonate (10 mg/mL), Boehringer Ingelheim Vetmedica GmbH, Ingelheim, Germany) or PRP (Angel System, Arthrex Vet Systems, Frechen, Germany; settings 5% HCT, 60 mL of blood, 3 mL PRP) was administered prior to incision closure but after the TPLO itself. The study was approved by the ethics committee of the faculty (No. 176–26-05-2019). The inclusion criteria were a body weight between 20 and 40 kg, CCLD based on clinical examination and preoperative magnetic resonance imaging, and no other concurrent orthopedic or neurologic disease. Dogs were excluded if they had a previous stifle joint infection or had undergone other intraarticular treatments, punctures, and injections within 4 weeks of surgery. Informed consent was obtained from the owner prior to participation in all cases. Dogs with bilateral cranial cruciate ligament disease were included in the study period, but only the second surgery was evaluated.

### 2.1 Surgical procedure, anesthesia, and analgesia

Based on the preanesthetic clinical exam and the resulting patient-specific American Society of Anesthesiologists (ASA) classification, a standard protocol for anesthesia and analgesia was used. This protocol consisted of premedication with 0.2 mg/kg diazepam (Solupam®, Dechra Veterinary Products Deutschland GmbH, Aulendorf, Germany), 2 mg/kg ketamine (Anesketin, Dechra Veterinary Products Deutschland GmbH, Aulendorf, Germany), and propofol for induction to effect (Narcofol, Fa. CP-Pharma Handelsgesellschaft mbH, Burgdorf, Germany), followed by endotracheal intubation and maintenance with isoflurane (Isoflurane CP®, Fa. CP-Pharma Handelsgesellschaft mbH, Burgdorf, Germany). Magnetic resonance imaging (MRI) was performed in addition to the clinical orthopedic examination to rule out concurrent meniscal injury (MAGNETOM Symphony 1.5 Tesla, Fa. Siemens Healthcare GmbH, Erlangen, Germany).

Two experienced surgeons (30 and 10 years of experience with TPLO) performed all TPLO surgeries, as described by Slocum and Slocum (1), aiming for a 5–6° postoperative TPA, but without using a jig. If a meniscal injury was found on MRI, patients underwent mini-arthrotomy caudal to the medial collateral ligament to confirm diagnosis and partial meniscal resection was performed (6). A standard commercial 3.5-mm or 3.5-mm broad TPLO plate (Depuy Synthes, Johnson & Johnson Medical GmbH, Norderstedt, Germany) was used. Locking screws were used in the TPLO plate head and one conventional cortical screw was placed in a compressed fashion within the plate shaft. The other screws were placed on surgeon preference with at least one other screw in a locking fashion in the plate shaft.

For peri- and postoperative analgesia and antibiotic medication, a standard protocol was used, which consisted of 0.2 mg/kg methadone (Comfortan®, Dechra Veterinary Products Deutschland GmbH, Aulendorf, Germany) intraoperatively and 0.015 mg/kg buprenorphine intravenously (Buprenovet® sine, Bayer Vital GmbH, Leverkusen, Germany) every 8 h for 1 day postoperatively, 4 mg/kg carprofen (Rimadyl; Zoetis GmbH, Germany) once daily for 10 days, and 20 mg/kg cefazolin intravenously (Cephazolin Fresenius 2 g; Fa. CP-Pharma Handelsgesellschaft mbH, Burgdorf, Germany) every 1.5 h intraoperatively and twice daily for 1 day, followed by oral administration (Cefatab, Fa. CP-Pharma Handelsgesellschaft mbH, Burgdorf, Germany) twice daily for 5 days was used. The dogs were discharged approximately 24 h postoperatively with the instructions of 6 weeks of rest and short on-leash walks.

### 2.2 Radiographs

Orthogonal radiographs of the affected stifle were obtained preoperatively, immediately after surgery, and 6 months postoperatively for the TPA measurements. Three independent observers measured the TPA values for each radiograph as described (1) using the integrated TPLO/TPA measurement tool provided within the imaging software dicomPACS® (Version 9.1.26, Oehm und Rehbein GmbH, Rostock, Germany). The values of each observer and the mean of all three observers were calculated for the TPAs preoperatively, immediately postoperatively, and 6 months postoperatively. The change in TPA was defined as the difference between the TPA values measured immediately postoperatively and at 6 months postoperatively, indicating TPA subsidence.

### 2.3 Effect on outcome

The association between TPA subsidence and clinical subjective lameness, LOAD score, and the outcome of objective gait analysis 6 months postoperatively was investigated. Therefore, all dogs underwent clinical examination by one observer with a subjective lameness score (score 0: no lameness, (1) mild intermittent lameness, (2) mild obvious lameness, (3) moderate lameness, (4) severe lameness). The gait analysis was performed using a force plate treadmill, which is equipped with four Kistler force plates (Spezialelemente Deutsche Sporthochschule Köln, Cologne, Germany) in combination with an optical system (Vicon Nexus Vicon Motion Systems Ltd., Oxford, United Kingdom, Quadruped Locomotion Software). The dogs had an acclimatization time of approximately 10 min to make them familiar with the treadmill. After acclimatization, the dogs walked on the treadmill while the speed was adjusted individually for each patient in 0.02 m/s increments. For an individual dog, the velocities were maintained constant at all evaluation times. The peak vertical force (PVF) and vertical impulse (VI) were evaluated in percent body weight. VI is defined as the integral of the vertical ground reaction force throughout the stance phase. The symmetry indices for the hind limbs of the PVF (SIPVF) and VI (SIVI) were calculated, as previously described (15). The validated LOAD questionnaire was performed by each owner. It was demonstrated previously that LOAD scores correlate with lameness and SIPVF (16, 17). A change of 4 points of the LOAD score was interpreted as a clinically significant difference (18). All outcome variables (lameness score, PVF, VI, SIPVF, SIVI, and LOAD score) were collected preoperatively, as well as at 6 weeks, 3 months, and 6 months postoperatively.

### 2.4 Statistical analysis

Data were collected in an Excel spreadsheet (Microsoft Excel, Microsoft Office, Redmond, US). For statistical analysis, SPSS (Version 28.0.1, IBM Corp, Armonk, New York, United States) was used. Data were tested for normal distribution using the Shapiro–Wilk and Kolmogorov–Smirnov tests. For normally distributed data, ANOVA or *t*-tests were used. Otherwise, non-parametric tests (Wilcoxon test, Friedman test, and Kruskal–Wallis test) were used. Interobserver reliability of TPA measurements was assessed using the intraclass correlation coefficient (ICC) and interpreted, as described by Cicchetti (19). Multiple linear regression was used to evaluate the impact of the independent variables (TPA subsidence, immediate postoperative TPA, 6-month postoperative TPA, meniscal injury, and whether the dog received PRP, HA, or no injection intraoperatively) on each of the dependent outcome variables PVF, VI, SIPVF, SIVI, and LOAD score. For assessing the impact of independent variables on lameness scores, a binary regression analysis was conducted, categorizing the animals as lame or non-lame 6 months postoperatively. Therefore, the dogs with a lameness score > 0 at 6 months postoperatively were categorized as lame. In total, six regression analyses were performed, one for each dependent variable. For each linear regression analysis, assumptions were verified as follows: linearity was checked using scatterplots, independence of errors using the Durbin–Watson statistic, homoscedasticity using scatterplots, normality of residuals using the Kolmogorov–Smirnov test, and multicollinearity using variance inflation factor values <10 and tolerance values >0.1. For the linear regression analysis, the coefficient of determination (*R*^2^) was interpreted following the guidelines as described by Cohen (20). For the binary regression analysis, the interpretation followed the methodology outlined by Backhaus et al. (21). Statistical significance was set at *p* < 0.05 for all tests.

## 3 Results

Two dogs were excluded from the analysis because radiographs for TPA measurement at the 6-month recheck post-op were not available. In total, 60 dogs were included in the study. Of these 60 dogs, 11 dogs were intact females, 21 were spayed females, 12 were intact males, and 16 were neutered males.

The mean age was 6.3 ± 3.2 years (range: 1–13 years). The mean bodyweight was 31.1 ± 6.5 kg (range: 20–40 kg). Breeds included mixed breed dogs (*n* = 17), Labrador Retriever (*n* = 15), Golden Retriever (*n* = 4), Doberman Pinscher (*n* = 4), Olde English Bulldog (*n* = 3), Rottweiler (*n* = 3), Beagle (*n* = 3), Swiss Shepherd (*n* = 2), and one of each Eurasier, Boxer, Entlebucher Mountain Dog, Bearded Collie, German Wachtel, Gordon Setter, Appenzeller Mountain Dog, Breton, and American Bulldog. A total of 25 dogs had concurrent meniscal damage in the affected stifle preoperatively. Eight dogs had developed contralateral cranial cruciate ligament disease within the study period, and only the second surgery was evaluated.

The results of the clinical examination, the gait analysis, and the LOAD scores are shown in Table 1. Treadmill speed during a walk ranged from 0.8 to 1.3 m/s for all dogs. As displayed in Table 1, all dogs showed significant improvements in all outcome variables of clinical, subjective lameness scores, LOAD scores, and the ground reaction forces measured using treadmill-based force plate gait analysis up to 6 months postoperatively. The LOAD score showed improvement for all dogs by a median of 13.5 scores from preoperative up to 6 months postoperative (*p* < 0.001), indicating a clinically significant improvement (18).

**Table 1:** Results of the clinical examination seen as lameness score median (range), results of the gait analysis during walk on the treadmill displayed as mean +/− standard deviation, results of LOAD score median (range), and *p*-values demonstrating improvement from preoperative up to 6 months postoperatively.

	Lameness score	PVF in % bodyweight	VI in % bodyweight	SIPVF	SIVI	LOAD score
Preoperative	3 (1–4)	30.09 ± 9.92	7.39 ± 2.83	42.71 ± 37.49	53.83 ± 43.87	35 (18–51)
6 weeks postoperative	1 (0–4)	33.95 ± 5.87	8.85 ± 2.21	21.50 ± 15.60	26.65 ± 15.60	28 (15–56)
3 months postoperatively	0 (0–4)	36.22 ± 6.15	9.40 ± 2.31	11.93 ± 11.41	13.57 ± 12.53	26 (13–48)
6 months postoperatively	0 (0–2)	39.21 ± 5.41	10.51 ± 2.37	6.86 ± 6.03	7.47 ± 6.64	21, 5 (13–40)
*p*-value	*p* < 0.001	*p* < 0.001	*p* < 0.001	*p* < 0.001	*p* < 0.001	*p* < 0.001

There were no statistically significant differences between the TPA measurements of the observers: preoperative (*p* = 0.08), immediately postoperative (*p* = 0.105), and 6 months postoperatively (*p* = 0.07). The ICC revealed excellent interobserver reliability for TPA measurements: preoperative ICC 0.92 (95% CI: 0.851–0.953), immediately postoperative ICC 0.84 (95% CI: 0.749–0.901), and 6-month postoperative ICC 0.86 (95% CI: 0.779–0.915). The intraarticular injection of PRP, HA, or the absence of injection demonstrated no significant differences in mean TPA subsidence (*p* = 0.74), mean immediate postoperative TPA (*p* = 0.23), or mean TPA at 6 months postoperatively (*p* = 0.43). As there were no differences between the observers and an excellent interobserver variability, the regression analysis and differences between TPAs immediately post-op and 6 months post-op were performed with the mean TPA values of the observers. A significant difference was found between the measurements of the mean TPA immediately postoperative and the mean TPA 6 months postoperatively (*p* = 0.015). The mean TPA subsidence was 0.22 ± 0.75°. The results of the TPA measurements and the calculated TPA subsidence are displayed in Table 2.

**Table 2:** Results of the tibial plateau angle (TPA) measurements and the resulting TPA subsidence in degrees displayed as mean +/− standard deviation.

	TPA preoperative	TPA postoperatively	TPA 6 months postoperatively	TPA subsidence
Observer 1	24.32 ± 3.13	4.86 ± 2.56	5.13 ± 2.59	0.25 ± 0.67
Observer 2	24.40 ± 3.05	5.97 ± 3.61	6.12 ± 3.50	0.15 ± 0.83
Observer 3	23.13 ± 3.37	6.13 ± 3.74	6.40 ± 3.36	0.27 ± 1.45
Mean of observers	23.95 ± 3.00	5.62 ± 2.94	5.88 ± 2.84	0.22 ± 0.75

The multiple linear regression analysis of PVF 6 months postoperatively indicated no significant associations and a low proportion of explained variance (*R*^2^ = 0.059; *p* = 0.668). Similarly, the analysis for VI 6 months postoperatively demonstrated no significant associations, with a moderate level of explained variance (*R*^2^ = 0.15; *p* = 0.121). For the LOAD score, the regression analysis revealed no significant associations, and the independent variables explained only a small proportion of the variance (*R*^2^ = 0.097; *p* = 0.361). In addition, the binary regression analysis for the lameness score at 6 months postoperatively showed no significant associations, with a small explanatory power (Nagelkerke’s *R*^2^ = 0.236; *p* = 0.137).

**Figure 2:**
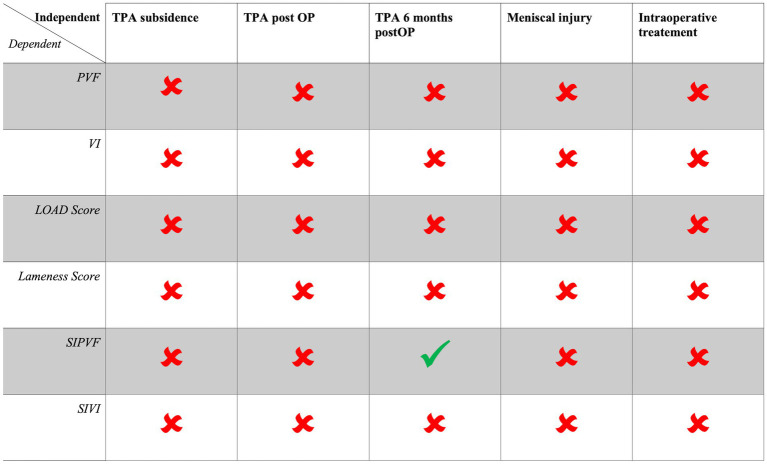
Summary of all multiple linear regression models, with dependent variables listed in the rows and independent variables presented in the columns. A checkmark (✔) indicates a significant independent predictor of the dependent variable (*p* < 0.05), while a cross (✖) denotes a non-significant independent variable (*p* > 0.05). The intraoperative treatment variable means if a dog received PRP, HA, or no injection intraarticular.

However, the multiple linear regression analysis model for the SIPVF at 6 months postoperatively was significant with a moderate explanation of variance (*R*^2^ = 0.210; *p* = 0.031). The 6-month postoperative TPA was the only significant predictor within the model (*ß* = 0.459; 95% CI: 0.41–1.44; *p* < 0.001), while other variables, such as TPA subsidence, immediate postoperative TPA, preoperative meniscal injury, and whether an injection was performed, showed no significant associations (*p* > 0.05). This indicates that with smaller TPA 6 months postoperatively, the dogs showed a more symmetrical gait regarding SIPVF. The analysis for the SIVI 6 months postoperatively revealed no significant associations and low explanatory power of the independent variables (*R*^2^ = 0.053; *p* = 0.742). Figure 2 summarizes all multiple linear regression models, highlighting the significant independent variables within each model.

## 4 Discussion

The results of this study showed that we must reject our hypothesis partially. None of the regression models demonstrated a relationship between TPA subsidence and the outcome variables (PVF, VI, SIPVF, SIVI, lameness score, and LOAD score), while controlling for other variables (postoperative TPA, 6-month postoperative TPA, meniscal injury, or the injection of PRP, HA, or no injection). Only for the SIPVF a positive association to the TPA six months postoperative was demonstrated showing that a lower TPA six months postoperative is associated with a more symmetrical gait in hindlimbs regarding SIPVF.

The obtained values for the preoperative TPA measurements are comparable to other studies of dogs with CCLD, ranging from 23.76° to 29° (3, 22, 23). In addition, the immediate postoperative TPA measurements are comparable to other studies, which evaluated TPA subsidence, ranging from 4.57 to 10.1° (9–11, 13). In contrast to other studies, we revealed a very small TPA subsidence of 0.22 ± 0.75° (Table 2), while other studies reported a TPA subsidence of 1.5 ± 2.2° (9), 1.9 ± 0.19° (10), 2.2 ± 2.7° (11), and 1.11 ± 3.5° (13). The use of hybrid locking TPLO plates in our study could explain why the measured TPA subsidence is lower than previously reported. Due to these previous reports, the use of locking plate–screw constructs influences the change in TPA and reduces or prevents TPA subsidence (10, 11). In addition, it is also questionable whether the additional locking screw in the plate shaft used in this study might have contributed to the smaller amount of TPA subsidence. It remains questionable whether the hybrid locking plates themselves or the screw configurations in the plate are a possible factor for the prevention of TPA subsidence. A prospective study comparing different plates and screw configurations would be needed to account for this question.

TPA measurements on radiographs themselves can be influenced by factors such as observer (3, 24), experience (24), limb positioning (25), or the degree of osteoarthritis (26), which must be seen as a limitation when measuring TPAs on radiographs. In addition, high interobserver variability was reported when assessing TPA measurements on radiographs (24, 26). To make our data more comparable to other data/studies and to avoid an effect as described by Caylor et al. (24), measurements were performed by three different observers. Interobserver variability was excellent for all taken measurements, and no significant differences were observed in this study.

The experience and technique of the surgeon performing TPLOs might be an additional factor that could lead to differences in postoperative TPA or TPA subsidence. This was not attributed in the current study and is a relative limitation as the TPLO is a strongly standardized procedure and only two surgeons with experience of ≥10 years in TPLOs performed the surgeries in this study. There are also studies showing that the surgeon’s experience has no influence on the outcome (13, 27) as well as on the TPA measurements postoperatively and at the recheck 42–84 days postoperatively (13).

In this population of dogs with CCLD, 25/60 (41.67%) had a concurrent meniscal injury prior to TPLO, which was managed with partial meniscal resection of the torn part during surgery. This amount of concurrent meniscal injury is comparable to the values from the literature with rates up to 84% (28). There was no association between concurrent meniscal injury and partial meniscectomy during stifle stabilization surgery on the outcome variables evaluated in this study, which is comparable to other studies using ground reaction forces as outcomes (29–31).

The TPA at 6 months postoperative showed an association with the SIPVF at 6 months postoperatively, demonstrating that dogs with a lower TPA at 6 months postoperative walk more symmetrically in the hindlimbs as this leads to lower values for SIPVF. This partially confirms the findings from the study by Wilson et al. (13), where the TPA measured at 42–84 days postoperatively significantly correlated with improvement in weight-bearing. They concluded that a lower TPA at the re-evaluation correlates with more improvement regarding weight-bearing in dogs after TPLO (13). However, it remains discussable why Wilson et al. (13) only showed a correlation for the improvement in weight-bearing and not for the symmetry index. In contrast, our study demonstrated a correlation between TPA and SIPVF, but not PVF or VI. One limitation and potential explanation by comparing our study to Wilson et al. (13) is that they used a stance analyzer, while we used a treadmill-based force plate gait analysis system. As our study and the study by Wilson et al. (13) indicated that it could be potentially beneficial to aim for lower TPAs, it has been proven that over-rotating the TPA below or over the recommended 5–6° showed no effect or difference on ground reaction forces at 4 or more months after TPLO for TPAs between 0 and 14° (12). However, some studies suggest that TPAs below 5° can be beneficial regarding craniocaudal stability of the stifle after TPLO (32) or meniscal load (33).

The limitations of this study include the study design with dogs receiving intraarticular injections of PRP and HA, which could impact the outcomes. In all multivariable regression analyses, the injection was considered a confounding variable, yet it showed no effect on the study results.In addition, PRP, HA, or no injection did not influence TPA measurements in this study. Moreover, a previous study involving all of the dogs analyzed in this study found no differences in outcomes between PRP, HA, or control groups at each evaluated recheck (6 weeks, 3 months, and 6 months postoperative) concerning clinical outcomes, objective force plate gait analysis, progression of osteoarthritis, or validated owner questionnaires (14). The second limitation is that the TPAs were only measured on radiographs while there is some evidence that 3D imaging methods contribute to a more precise measurement (3). Third, this study included dogs with both unilateral and bilateral CCLDs, so the SIPVF and SIVI were calculated to account for the involvement of both hindlimbs. However, it is important to consider that load shifting to the forelimbs may have influenced the results. Given the frequent bilateral occurrence of CCLD (34), this reflects the nature of CCLD and similar clinical studies have included bilateral cases (7, 14). To minimize potential data bias, only the second surgery was included in the analysis for dogs with bilateral CCLD, based on the assumption that the first stifle provided adequate stability. A previous study involving these same dogs showed no postoperative differences in the measured outcome parameters (GRF, lameness score, and LOAD score) (14), suggesting that the inclusion of bilateral cases may be a relative limitation. Fourth, it remains open if TPA subsidence is smaller or not evident while using locking TPLO plates with at least one locking screw in the TPLO plate shaft, and the postoperative TPA stays more constant. This question cannot be answered in this study and should be addressed in further prospective, comparative studies with different screw and/or plate configurations. Another limitation is that the time when measuring TPA subsidence differs between studies, making comparisons difficult. For example, Moeller et al. (9) performed recheck radiographs 28–65 days postoperative, Wilson et al. (13) re-evaluated the dogs between 42 and 84 days after surgery, Conkling et al. (10) performed the re-evaluation 8 weeks postoperative, and Krotscheck et al. (11) had a median time of 75 days for the re-evaluation with up to 2,815 days. Therefore, the change in TPA and the effect on outcome after 6 months in our study might differ from those reported in other studies. It is possible that the TPA will change over time, and further longitudinal studies are needed. However, the author believes that after fibrous formation of the callus, little to no change in TPA is anticipated.

In conclusion, the study indicated that TPA subsidence is not associated with the postoperative outcome 6 months postoperatively after TPLO. The amount of TPA subsidence is potentially smaller when using newer TPLO locking implants and at least one locking screw in the plate shaft. Lower TPAs at 6 months postoperatively are associated with a more symmetrical gait in hindlimbs as measured by SIPVF 6 months postoperatively; this confirms the findings of the study by Wilson et al. (13).

## Data Availability

The raw data supporting the conclusions of this article will be made available by the authors, without undue reservation.
